# Mica Lattice Orientation of Epitaxially Grown Amyloid β25–35 Fibrils

**DOI:** 10.3390/ijms251910460

**Published:** 2024-09-28

**Authors:** György G. Ferenczy, Ünige Murvai, Lívia Fülöp, Miklós Kellermayer

**Affiliations:** 1 Department of Biophysics and Radiation Biology, Semmelweis University, Tűzoltó u. 37-47, 1094 Budapest, Hungary; ferenczy.gyorgy@semmelweis.hu (G.G.F.); murvai.unige@semmelweis.hu (Ü.M.); 2 Department of Medical Chemistry, University of Szeged, Dóm tér 8, 6720 Szeged, Hungary; fulop.livia@med.u-szeged.hu

**Keywords:** amyloid β, mica surface, epitaxial growth, nanotechnology, atomic force microscopy, molecular dynamics

## Abstract

β-amyloid (Aβ) peptides form self-organizing fibrils in Alzheimer’s disease. The biologically active, toxic Aβ25–35 fragment of the full-length Aβ-peptide forms a stable, oriented filament network on the mica surface with an epitaxial mechanism at the timescale of seconds. While many of the structural and dynamic features of the oriented Aβ25–35 fibrils have been investigated before, the β-strand arrangement of the fibrils and their exact orientation with respect to the mica lattice remained unknown. By using high-resolution atomic force microscopy, here, we show that the Aβ25–35 fibrils are oriented along the long diagonal of the oxygen hexagon of mica. To test the structure and stability of the oriented fibrils further, we carried out molecular dynamics simulations on model β-sheets. The models included the mica surface and a single fibril motif built from β-strands. We show that a sheet with parallel β-strands binds to the mica surface with its positively charged groups, but the C-terminals of the strands orient upward. In contrast, the model with antiparallel strands preserves its parallel orientation with the surface in the molecular dynamics simulation, suggesting that this model describes the first β-sheet layer of the mica-bound Aβ25–35 fibrils well. These results pave the way toward nanotechnological construction and applications for the designed amyloid peptides.

## 1. Introduction

Amyloid fibrils are nanoscale protein filaments that can be found in the extracellular tissue deposits of various degenerative disorders ranging from Alzheimer’s disease to type 2 diabetes [1,2,3,4]. Even though amyloid peptides are toxic, they have been suggested for uses in various nanotechnological applications because they self-assemble into specific nanostructures such as nanosensors and conductive nanowires [5,6,7,8]. A special advantage of amyloid peptides is that variants suitable for a particular application may be generated by chemical [9] or biotechnological methods [8]. Once the fibrils are formed, they possess high structural stability under relatively harsh physical and chemical conditions. The global disorder in amyloid fibrillar arrangement, however, stands in the way of their widespread usability in nanotechnology applications. In the present work, we investigated amyloid beta 25–35 (Aβ25–35), a fragment of the Alzheimer beta-peptide (Aβ-peptide). Alzheimer’s disease is a neurodegenerative disorder characterized, among others, by the deposition of insoluble filamentous aggregates called neuritic plaques [10,11]. The 39- to 43-residue-long Aβ-peptide is the major component of neuritic plaques, and it forms self-associating fibrillar structures possessing predominantly cross-β conformation [4]. Aβ25–35 is a fragment of the full-length Aβ-peptide comprising a stretch of residues from amino acid positions 25 to 35. Aβ25–35 is thought to be the biologically active fragment of Aβ, considering that it forms fibrils and retains the toxicity of the full-length peptide [12,13,14]. The exact mechanisms of amyloidogenesis and fibrillogenesis, and the molecular structure of β-amyloid fibrils, are not clearly understood at present. It is thought that the basic features of the Aβ25–35 fibril are similar to those formed from other peptides. Thus, adjacent peptides are connected to each other via hydrogen bonds along the axis of the protofilament so as to form a β-sheet, and amino acid side chains lie perpendicular to the axis.

It has been shown that charged surfaces interact with Aβ25–35 [15,16], suggesting that its binding and, possibly, orientation may be controlled by electrostatic mechanisms. We have previously investigated the formation of Aβ25–35 fibrils on the negatively charged mica surface by using atomic force microscopic (AFM) methods. Mica is a phyllosilicate, in which silicon (Si), aluminum (Al), phosphorus (P), and oxygen (O) atoms are organized into crystalline planar layers, which are held together by potassium ions (K^+^) [17,18,19]. On the cleavage surface of mica (plane perpendicular to the [001] direction), hexagonally ordered O atoms form K^+^-binding pockets. In freshly cleaved mica exposed to aqueous buffer solutions, K^+^ diffuses away depending on the association–dissociation kinetics, and thus an overall negatively charged surface is displayed, onto which biomolecules may adhere. In the past, the oriented self-organization of various biomolecular systems on mica has been demonstrated [20,21]. We have found that Aβ25–35 fibrils display a highly ordered trigonal arrangement on mica, and the binding is very sensitive to K^+^ ions [22]. By using acetylated peptides, we have shown that Lys28, and to a lesser degree the N-terminus, play determinant roles in oriented filament formation, indicating that positively charged regions of the peptide compete with K^+^ for surface binding [23]. Time-lapse AFM imaging revealed that oriented fibril formation is the result of epitaxial filament growth rather than the binding of already-assembled fibrils from solution [24]. Furthermore, the epitaxial growth of Aβ25–36 fibrils displays stepwise dynamics, suggesting that a slight mismatch in the periodicities of the lattice and the fibrils may play a role in growth dynamics [24]. According to AFM and FTIR spectroscopic measurements, the oriented Aβ25–35 fibrils possess a β-sheet structure and are made up of one to four β-sheets [25].

Even though the epitaxial growth of Aβ25–35 fibrils on mica is clearly dictated by the atomic structure of mica’s surface lattice, it is currently unknown exactly which symmetrical axis of the lattice corresponds to the fibril orientation. Furthermore, it is unclear whether the consecutive β-strands in the β-sheets of the oriented Aβ25–35 fibrils are ordered in parallel or antiparallel with respect to one another. To answer these questions, we performed high-resolution AFM measurements combined with molecular dynamics simulations of model peptides. Our results suggest that antiparallel β-sheets of Aβ25–35 fibrils are oriented along the long diagonal of the oxygen hexagon in the surface lattice of mica.

## 2. Results and Discussion

**Figure 1:**
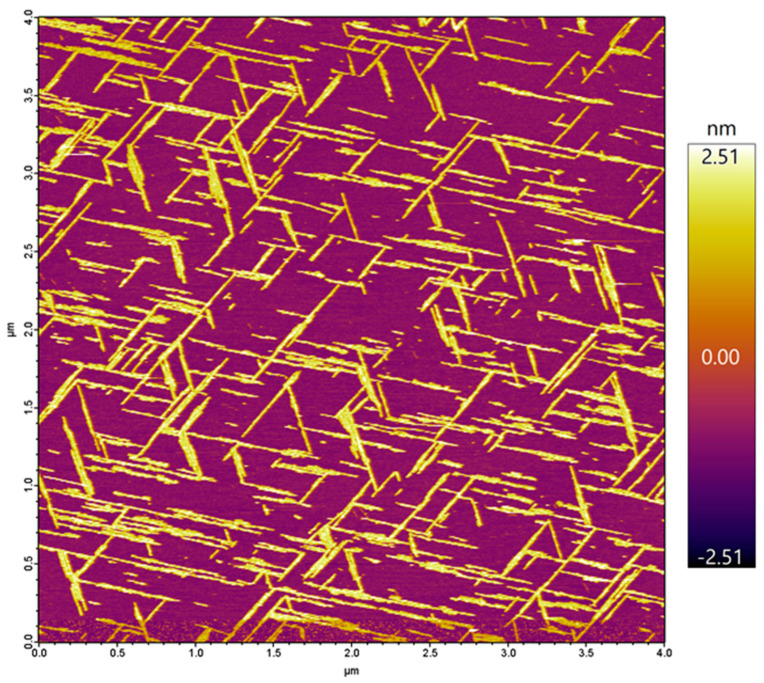
Height-contrast AFM image of a mica surface on which Aβ25–35 fibrils were grown by epitaxy. The fibrils are of a more-or-less uniform width and height, straight, and are oriented in three main directions that reflect the underlying hexagonal lattice structure of mica. Color bar indicates the topographical height scale of the AFM image.

**Figure 2:**
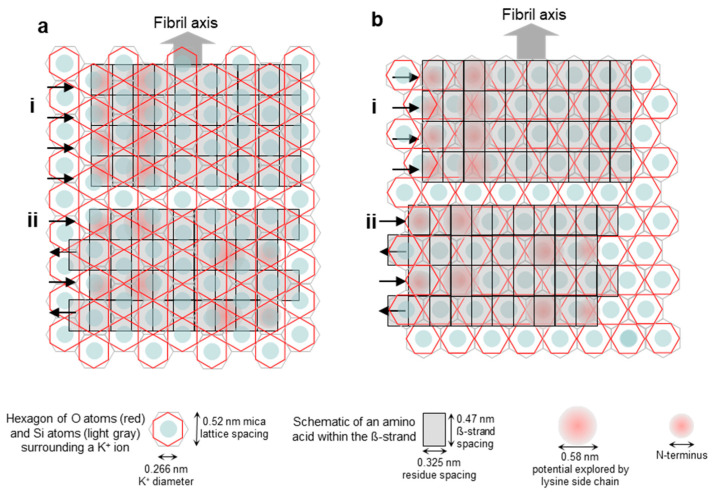
Schematics of the possible arrangements of the epitaxially grown Aβ25–35 fibrils on the mica surface with respect to the mica lattice. (**a**) Fibril axis is parallel to the edge of the oxygen-atom hexagon (i.e., oriented along its long diagonal) and hence perpendicular to the edge of the silicon-atom hexagon. (**i**,**ii**) Parallel and antiparallel β-strand orientations, respectively. (**b**) Fibril axis is perpendicular to the edge of the oxygen-atom hexagon (i.e., oriented along its short diagonal) and hence parallel with the edge of the silicon-atom hexagon. (**i**,**ii**) Parallel and antiparallel β-strand orientations, respectively. Altogether, the structural scenarios described in the text are as follows: (**ai**) scenario 1, (**aii**) scenario 2, (**bi**) scenario 3, and (**bii**) scenario 4.

In the present work, we investigated the topology of the oriented Aβ25–35 network with respect to the atomic crystal lattice structure of mica. The overall topology of the Aβ25–35 fibril network, grown from peptides with an epitaxial mechanism, is shown in Figure 1. The fibrils display three main orientations separated by 120°, which altogether correspond to the axes of a regular (equilateral) hexagon. The highly oriented and regular nature of the epitaxially evolved Aβ25–35 fibrils is in stark contrast to the twisted and curved topology of Aβ25–35 fibrils grown in solution, as we have documented earlier [25]. We have characterized the properties of the oriented Aβ25–35 fibril network in several prior studies, which revealed that it displays the spectroscopic signatures of β-sheets [25]; 1–4 β-sheets on top of each other contribute to a single fibril [25]; the fibrils grow epitaxially on the surface of mica with stepwise dynamics [24]; and peptide binding to mica and fibril growth may be inhibited by increasing the free K^+^ ion concentration [22]. Altogether, the topology of the epitaxially grown Aβ25–35 fibril network is dictated by the hexagonal lattice structure of mica, similarly to the oriented organization of other organic molecular systems on structured crystalline surfaces [26,27,28]. We note here that while the binding of the first β-sheet of the fibril is indeed dictated by the properties of the mica lattice and the side-chain features of the peptide, how exactly the subsequent β-sheets associate with the underlying ones still requires investigation. In the case of Aβ25–25, the epitaxial growth is deterministically influenced by its positively charged groups, the ε-amino group of Lys28 and the amino terminus [23]. However, there are two main ways in which the Aβ25–35 fibrils may be oriented with respect to the hexagonal mica lattice. One possibility is that the fibrils are oriented along the long diagonals of the oxygen hexagon. Alternatively, they may be oriented along the short ones. The two topologies, while macroscopically identical, differ from each other by 30° with respect to the mica lattice. We consider the oxygen (O) hexagons of the mica lattice for reference, because they are the ones positioned on the surface of the cleaved mica. We note, however, that the chemically and structurally important, underlying silica (Si) hexagons are oriented at 30° with respect to the O hexagons (see Figure 2). Thus, the first question we investigated was which orientation is followed by the epitaxially evolved Aβ25–35 fibril network. The second prevailing question was whether the β-sheets of the Aβ25–35 fibrils are composed of parallel or antiparallel β-strands. To answer these questions, we carried out atomic-resolution AFM measurements and molecular dynamics simulations. 

To address the problem of orientation and consider the possible scenarios, we first generated an empirical visual model of an Aβ25–35 fibril β-sheet on the mica lattice (Figure 2). Four scenarios are shown. In the first (Figure 2(ai)), a β-sheet composed of parallel β-strands is oriented along the long diagonal of the hexagon of oxygen atoms, which are on the surface of mica. This orientation is parallel to the edges of the oxygen hexagon and it is perpendicular to the edge of the hexagon of silicon atoms that lie just underneath the oxygens. In the second scenario (Figure 2(aii)), a β-sheet composed of antiparallel β-strands is oriented in the same way as in Figure 2(ai). In the third scenario (Figure 2(bi)), a β-sheet composed of parallel β-strands is oriented along the short diagonal of the oxygen hexagon, and in the fourth scenario (Figure 2(bii)), a β-sheet composed of antiparallel β-strands is oriented in this same direction. Because the positive moieties of the Aβ25–35 β-sheet are responsible for binding to the mica surface at the K^+^-binding pockets, the greater the overlap between them, the stronger the binding. This simple empirical scheme suggests that scenario 4 (Figure 2(bii)) might be the most likely, followed by scenarios 1, 2, and 3 (in this order). We note, however, that this simple scheme does not take into account factors such as the conformational distribution of the lysine and the N-terminal residues and the vertical distance between the charges and the K^+^-binding site, although they may modulate the arrangement.

**Figure 3:**
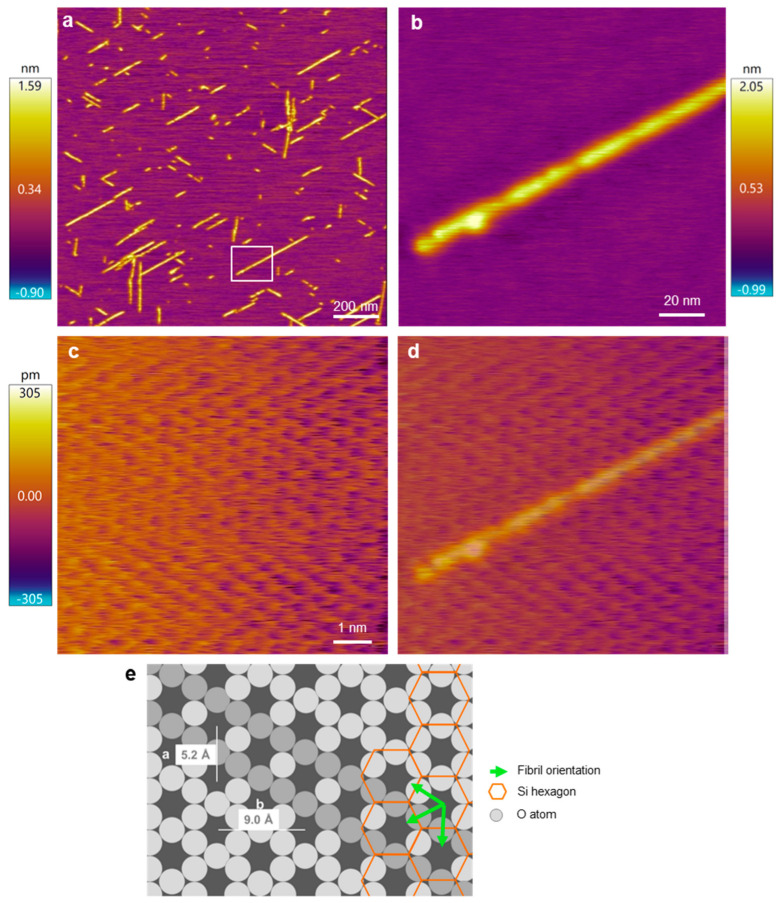
AFM analysis of the Aβ25–35 fibril orientation with respect to the mica crystal lattice. (**a**) Overview height-contrast AFM image of Aβ25–35 fibrils. (**b**) Magnified AFM image of the area boxed in (**a**). (**c**) Atomic-resolution AFM image of a 10-by-10 nm area in the sample. (**d**) Overlay of images (**b**,**c**), for a better comparison of the fibril and lattice orientations. (**e**) Schematics of the hexagonal mica structure with the overlain Aβ25–35 fibril orientations. The lattice orientation in the schematic diagram is essentially identical to that in the AFM image. Accordingly, the fibril axis is parallel to the oxygen-atom hexagon and hence perpendicular to the edge of the silicon-atom hexagon. Fibril orientations are shown by green arrows and a selected orientation is indicated by a row of more darkly shaded O atoms. Color bars indicate the topographical height scales of the AFM images.

To experimentally test which of the orientation scenarios occurs, β25–35 fibrils on the mica surface were investigated with AFM by comparing the fibril directions and the atomic resolution image of the mica surface (Figure 3). Straight, oriented fibrils with a smooth surface could be observed (Figure 3a,b). To identify the mica lattice orientation, we zoomed into a small, 10-by-10 nm region in between the oriented fibrils (Figure 3c). The lattice structure and rows of the crystal unit cell could be well resolved. To compare the orientation of the lattice with that of the Aβ25–35 fibrils, we superimposed the two images (Figure 3d) and found that the two exactly coincide. Because the unit cells correspond to the oxygen hexagons, the Aβ25–35 fibril is oriented along its long diagonal. To circumvent the problem of thermal drift, we carried out AFM scanning in both directions (up and down) and received similar results. As such, based on the AFM images, the Aβ25–35 fibrils are oriented on the mica surface according to either scenario 1 or 2 (Figure 2(ai,aii)). 

**Figure 4:**
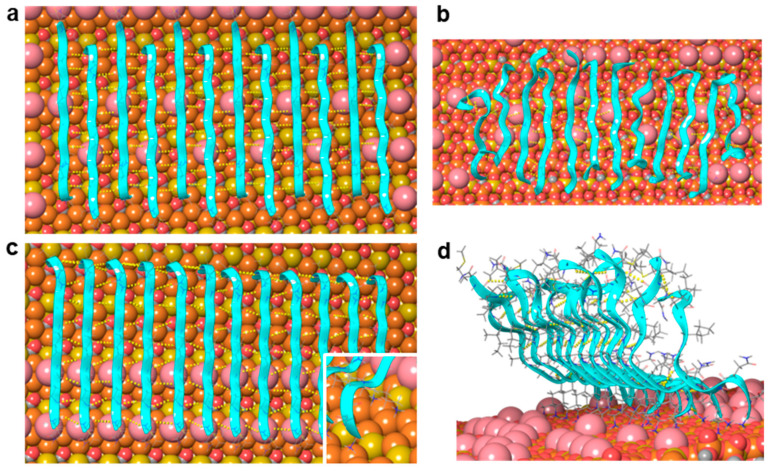
Molecular dynamics simulation of a twelve-strand-long, single-layer Aβ25–35 β-sheet on mica. (**a**,**b**) Initial and final arrangements of an antiparallel β-sheet after a 100 ns long simulation, respectively. (**c**,**d**) Initial and final arrangements of a parallel β-sheet after a 100 ns long simulation, respectively. Mica is shown here with a space-filling model: Si coral, Al mustard, O red, K pink. Peptide strands are shown with ribbons and in thin tube representation. Backbone hydrogen bonds are indicated with yellow dotted lines.

To test whether the β-sheet of the oriented Aβ25–35 fibril is composed of parallel or antiparallel β-strands, we performed molecular dynamics simulation on model peptides (Figure 4). Two models of a single-layer amyloid β-sheet bound on the mica surface were built. One model was constructed from antiparallel and the other one from parallel β-strands. The strands lay orthogonally to the fibril direction. To mimic the experimental results, the fibril direction was adjusted along the long diagonal of the oxygen hexagons, perpendicular to the side of the underlying silica hexagons. The N-terminal charged amino groups and the charged ε-amino groups of Lys28 were brought in contact with the mica surface by replacing K^+^ ions. The initial models of the molecular dynamic (MD) simulations included regular sheets parallel to the mica surface except the N-terminal of the strand, which leaned toward the K^+^ binding site (Figure 4a,c). These models were in accordance with the available experimental data. They were composed of β-sheets in line with the FTIR spectrum recorded previously [25], and the sheets were parallel to the mica surface, as was suggested by force spectroscopy results [29]. The fibril direction was perpendicular to the edges of the Si hexagons (and, hence, in parallel with the main axis of the O hexagons), the 5.2 Å periodicity of which agreed with the separation of adjacent hydrogen-bonded β-strands (Figure S1a) and was close to the optimal separation of strands in antiparallel β-sheets [30]. The charged amino groups of Lys28 of the antiparallel strands fit in the K^+^ binding sites of the mica surface (Figure S1b), and this was consistent with the observed sensitivity of the fibril growth rate to the K^+^ concentration [22]. The Asn27 side chain was oriented upward, which was in line with the accessibility of the cysteine residue of the Aβ25–35 N27C mutant [31,32].

We must note that the optimal separation of parallel β-strands is near to 4.8 Å [30], while the periodicity of the mica surface in the fibril direction was 5.2 Å (Figure 2 and Figure 3). We speculate that the small difference between these values does not prevent the formation of β-sheets on the mica surface, but may lead to stress as the amyloid fibril is extending, and hence to the stepwise dynamics of epitaxially growing fibrils observed earlier [24].

The sheet with antiparallel strands (corresponding to scenario 2, Figure 2(aii)) remained quasi-parallel to the mica surface during two independent 100 ns MD simulations (Figure 4b, Video S1). The charged amino groups typically kept their positions in the K^+^-binding sites, although occasionally they jumped into neighboring sites. The number of H-bonds among the peptide backbones stabilized after a few nanoseconds at 39 ± 3 (Figure S2), which corresponds to 3–4 H-bonds between two strands. The strands at the edges of the sheet exhibited the largest mobility and deviation from the β-sheet structure, which was in accordance with their asymmetric environment and weaker stabilization by H-bonds.

The sheet with parallel strands (corresponding to scenario 1, Figure 2(ai)) behaved differently. The charged N-terminal Gly25 residues and the charged ε-amino groups of Lys28 remained bound to the surface, but the C-terminal end moved away from the mica surface and adopted an upward orientation (Figure 4d, Video S2). The parallel strands, however, remained largely H-bonded both at the N-terminal close to the mica surface and also at the C-terminal separated from the surface by several Ångströms. The number of H-bonds among the backbones was fairly constant after a few nanoseconds and took the value of 57 ± 3 (Figure S2), which corresponds to ~5 H-bonds between two strands. This number exceeds the average number of H-bonds (between 3 and 4) observed for the antiparallel sheet. The strands at the edges of the sheet are the most mobile ones, just as in the case of the antiparallel sheet. The simulation results do not exclude the possibility that parallel β-strands may be a stable arrangement on the mica surface, because the charged N-terminal and Lys28 side chain appeared firmly bound to the mica surface, and the strands remained H-bonded during the 100 ns simulation. However, the observed drifting of the C-terminal away from the mica surface leads to a fibril height of more than 20 Å, which far exceeds the ~1 nm thickness of the first fibril layer observed in AFM images [25]. 

**Figure 5:**
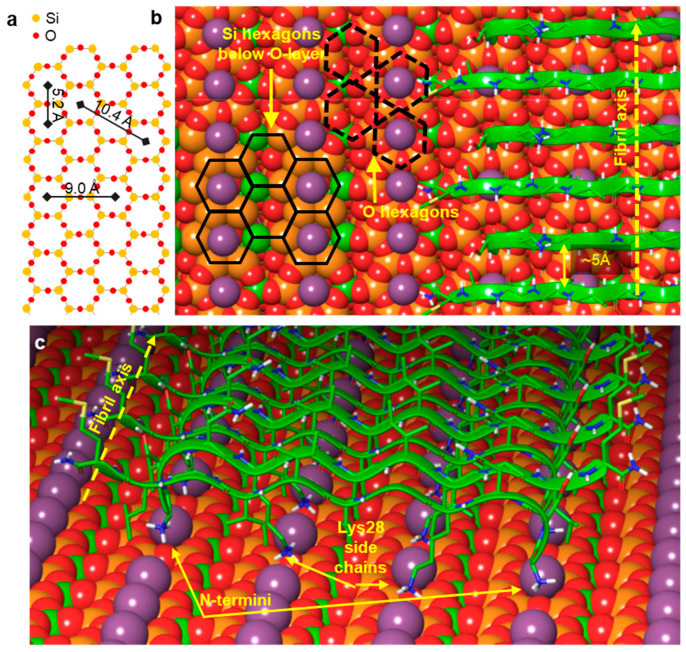
Summary of the most plausible structural arrangement of the epitaxially grown Aβ25–35 fibril on mica. (**a**) Hexagonal arrangement of the oxygen and silicon atoms in the surface crystal lattice of mica. (**b**) Section of an Aβ25–35 fibril. The fibril axis orientation is indicated with a yellow dotted arrow. Selected O-hexagons are indicated by black dashed lines and selected Si-hexagons by black solid lines. (**c**) Perspective view of the first β-sheet of the Aβ25–35 fibril, in which the β-strands are oriented antiparallel.

The simulation results imply that sheets composed of a large number of antiparallel strands are stable, preserve their periodicity along the fibril direction, and remain bound to the mica surface, in accordance with the experimentally observed epitaxial filament formation. Our model includes a single layer of β-sheet bound to the mica surface by polar interactions. The maximum height of the model is estimated to be ~11 Å (Figure 5 and Figure S3), and this is consistent with the observed height distribution of the fibrils, which suggests the presence of one to four layers on top of each other [25]. The height distribution shows a low frequency of single layers and a preference for fibrils with two or three layers. Since the β-sheets composed of Aβ25–35 strands are dominantly hydrophobic, they may associate to form multiple layers, with an increased stability compared to a single layer. While the exact mechanisms behind the vertical (i.e., perpendicular to the mica surface) stacking of β-sheets, and hence the thickening of Aβ fibrils, need to be investigated further, the results obtained in this work indicate that the fundamental first step during the epitaxial assembly of Aβ25–35 fibrils is the formation of an antiparallel β-sheet oriented along the long diagonal of the oxygen hexagons of the mica lattice. The findings lend the possibility of tuning the epitaxial Aβ25–35 assembly, so that fibril networks tailored to different nanobiotechnological applications may be devised. 

## 3. Materials and Methods

### 3.1. Sample Preparation

Aβ25–35 (^25^GSNKGAIIGLM^35^-amide) was produced by solid-state synthesis [9]. The lyophilized peptide was dissolved in dimethyl sulfoxide (DMSO, 50.0 mg/mL) and transferred to Na-phosphate-buffered saline (Na-PBS) buffer [10 mM Na-phosphate (pH 7.4), 140 mM NaCl, 0.02% NaN_3_]. Insoluble aggregates were removed by high-speed centrifugation at 250,000× *g* at 4 °C for 2 h (Beckman Coulter Optima^TM^ MAX Ultracentrifuge, Brea, CA, USA). The final concentration ranged between 0.5 and 1 mg/mL. Aliquots of the sample were quickly frozen in liquid nitrogen and stored at −80 °C for further use. Supernatant was diluted for further use with buffer to a typical working concentration of 5 µM. The peptide concentration was measured with a quantitative bicinchoninic acid assay [33].

### 3.2. Atomic Force Microscopy

Typically, 50 μL of sample was applied to a freshly cleaved mica disk surface (diameter 1 cm). We used high-grade mica sheets (V2 grade, #52-6, Ted Pella, Inc., Redding, CA, USA). After 10 min of incubation, the mica surface was washed with de-ionized water to remove the unbound fibrils and dried with high-purity N_2_ gas. The samples were imaged in air. Contact and hybrid-mode AFM images were acquired with an Asylum Research Cypher AFM instrument (Santa Barbara, CA, USA) with OTESPA (Bruker, Camarillo, CA, USA) or Arrow UHFAuD cantilevers (NanoWorld, Neuchatel, Switzerland). We collected 512 × 512-pixel images at a typical line-scanning frequency of 20–40 Hz and with a set point of 150 mV.

### 3.3. Image Processing and Data Analysis

AFM images were analyzed using algorithms built in the Asylum Research controller software v13.16.100 (Santa Barbara, CA, USA) running under IgorPro v6.03 (Wavemetrics, Lake Oswego, OR, USA). 

### 3.4. Molecular Dynamics Simulation

A single-layer mica model was built with a KAl_2_(Si_3_Al)O_10_(OH)_2_ composition according to the Loewenstein Al avoidance rule [34], with the fluid-contact-mode AFM coordinates of Kuwahara [35], using the program VESTA [36]. Aβ25–35 (^25^GSNKGAIIGLM^35^-amide) peptide beta strands were built, and β-sheets of 12 strands were constructed both with parallel and antiparallel strands. The sheets were positioned on the 80 Å × 80 Å mica surface. K^+^ ions were removed at positions where N-terminal amino and lysine ε-amino groups were in contact with the mica surface. The sheets were built and positioned on mica using Maestro [37]. The peptide–mica system was solvated with a box of ~11,000 TIP3P [38] water molecules, and ions were added to ensure neutrality using VMD [39]. Molecular dynamics simulations were performed with the NAMD software v2.11b1 [40]. The CHARM22 force field [41] was used for the peptide, and CLAYFF [42] for the mica, and interaction terms were obtained using the Berthelot mixing rules [43]. A 12 Å cutoff with smoothing from 10 Å was used for van der Waals interactions, and the particle mesh Ewald method [44] was applied for long-range electrostatics. The system was first minimized for 10,000 steps with fixed peptides and mica and for an additional 10,000 steps, allowing the whole system to freely relax. The system was then heated to 300 K in 30 ps and equilibrated for 250 ps at 1 atm, and 300 K. 2 fs time step was applied in all stages of the simulations. NPT equilibration of 500 ps and NVT equilibration of 200 ps were followed by 100 ns NVT production runs. The simulation with an antiparallel β-sheet structure was repeated with identical parameters but with a different initial velocity distribution.

The simulation results were evaluated by Maestro [37] and VMD [39]. H-bonds were analyzed by using a 3.5 Ångström donor–acceptor distance and 35° acceptor–donor-H angle as the upper limits for H-bonds.

## 4. Conclusions

Aβ25–35 forms an oriented network of epitaxially grown fibrils on mica surfaces. The fibrils adopt a straight form, in contrast to helical fibrils and bundles grown in solution. AFM images reveal that the fibril direction is along the long diagonal of the oxygen hexagon, which is perpendicular to the edges of the underlying silicon hexagons of the mica lattice. An atomic model of the first layer was built with antiparallel β-sheets. The structural characteristics of the model were preserved in molecular dynamics simulations. The model geometry is in line with the mica periodicity, fibril orientation, and height. Binding is dominated by the electrostatic interactions between the K^+^ sites of the mica surface and the positively charged N-terminus and Lys28 ε-amino groups of the peptides. The modeled first layer is proposed to associate with further β-sheets primarily by hydrophobic interactions to form multiple layers on the surface. The slight mismatch between mica-lattice periodicity and β-strand spacing in relaxed β-sheets may be responsible for the stepwise dynamics of epitaxial Aβ25–35 fibril growth observed previously [24]. Altogether, our findings lead us to propose that the oriented amyloid fibril network obtained by epitaxial mechanisms may be utilized in a number of nanobiotechnological applications.

## Data Availability

Data is contained within the article or Supplementary Material.

## Supplementary Materials

The following supporting information can be downloaded at https://www.mdpi.com/article/10.3390/ijms251910460/s1.
