# Supplementary material for: Mica Lattice Orientation of Epitaxially Grown Amyloid β25–35 Fibrils
Source: Int J Mol Sci. 2024 Sep 28;25(19):10460. doi: 10.3390/ijms251910460 (PMC11476711; doi:10.3390/ijms251910460)
Supplement: Supplementary file 1 [file ijms-25-10460-s001.zip › SI_20240920.pdf]

Supplementary Material

Lattice orientation of epitaxially grown amyloid ß25-35 network

György G. Ferenczy, Ünige Murvai, Lívia Fülöp[1] and Miklós Kellermayer*

Department of Biophysics and Radiation Biology, Semmelweis University, Tűzoltó u. 37-47, Budapest, 1094 Hungary

[1]Department of Medical Chemistry, University of Szeged, Dóm tér 8., Szeged, H-6720 Hungary

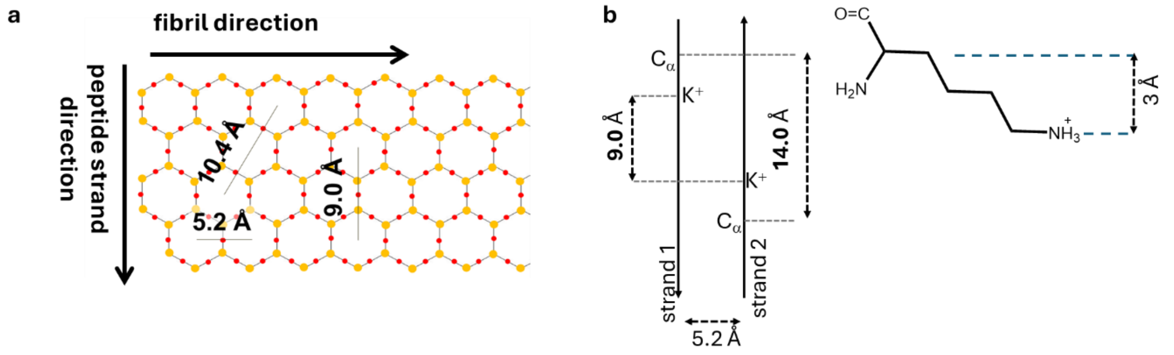

Figure S1. a. Schematic representation of mica with characteristic distances together with peptide and fibril directions of the atomic models. Si-hexagons are shown after the removal of surface O-atoms. Color code: O - red, Si – orange. b. Lysine Cα atoms of adjacent antiparallel strand are separated by 14 Å along the strand direction, but the flexibility of the sidechains allows the binding of the terminal protonated amine to the K^+ binding sites separated by 9 Å.

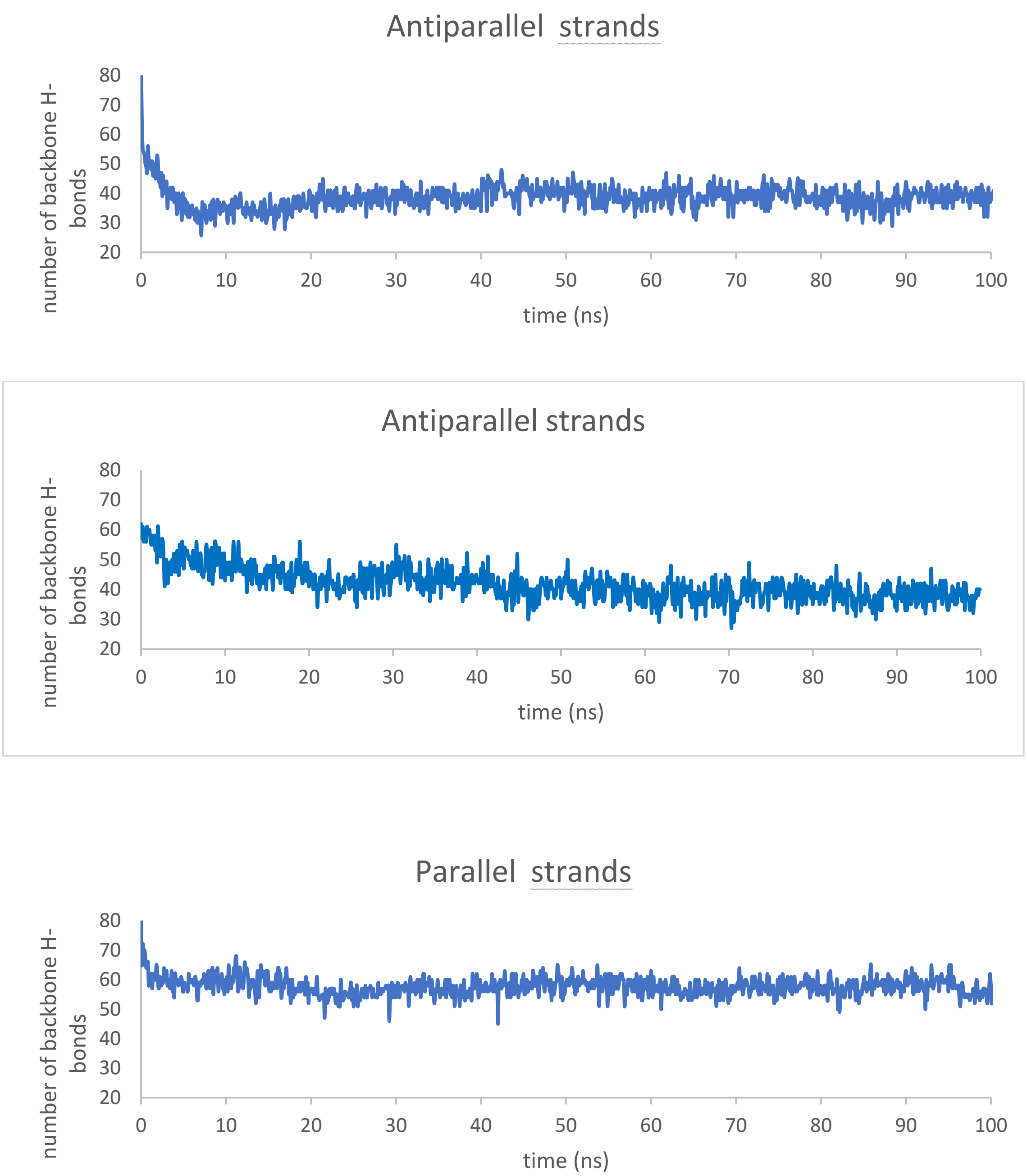

Figure S2. Number of backbone hydrogen bonds in the molecular dynamics simulations for the models with antiparallel (two independent simulations) and parallel strands.

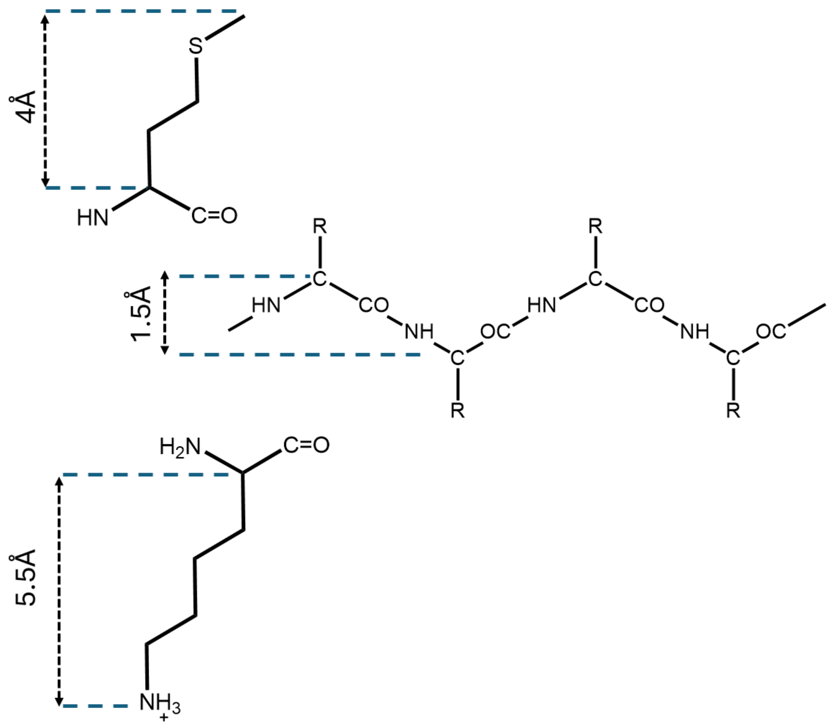

Figure S3. The 11Å maximal height of the Aβ-25-35 fibril is estimated as the sum of the heights of Lys28 (5.5Å), the β-pleated sheet (1.5Å) and Met35 (4Å).

Supplementary Video S1. 100-ns-long video about the molecular dynamics simulation of the anti-parallel Aß25-35 ß-sheet on mica. H-bonds are indicated as yellow segmented lines. Color codes: Si – yellow, Al – pink, O – red, K^+ Olive

Supplementary Video S2. 100-ns-long video about the molecular dynamics simulation of the parallel Aß25-35 ß-sheet on mica. H-bonds are indicated as yellow segmented lines. Color codes:
